# Biomechanical effects of root/cortical bone relation on tooth movement during premolar-extraction space closure with clear aligners: a finite element study

**DOI:** 10.3389/fbioe.2025.1717813

**Published:** 2025-11-19

**Authors:** Yihan Dong, Ya Wang, Zun Yang, Weiyi Gong, Man Guo, You Wu, Yun Hu, Leilei Zheng

**Affiliations:** 1 The Affiliated Stomatological Hospital of Chongqing Medical University, Chongqing, China; 2 Chongqing Key Laboratory of Oral Diseases, Chongqing, China; 3 Chongqing Municipal Key Laboratory of Oral Biomedical Engineering of Higher Education, Chongqing, China; 4 Chongqing Municipal Health Commission Key Laboratory of Oral Biomedical Engineering, Chongqing, China; 5 The Fifth People’s Hospital of Chongqing, Chongqing, China

**Keywords:** finite element study, clear aligner, root/cortical bone relation, sagittal rootposition, tooth movement, space closure

## Abstract

**Introduction:**

This study aimed to evaluate the biomechanical effects of varying sagittal root position (SRP), root length (RL), and cortical bone thickness (CBT) on tooth movement and stress distribution during clear aligner therapy (CAT) in extraction cases, using finite element analysis.

**Methods:**

Three-dimensional finite element models, including the maxillary alveolar bone, periodontal ligament (PDL), dentition, and clear aligner, were constructed. Groups with varying SRP (labial, middle, and palatal), RL (long, normal, short) and CBT (1 mm, 2 mm) were established. Tooth movement and stress distribution were analyzed for each group after 0.2 mm anterior tooth retraction.

**Results:**

Without attachments or additional forces, clear aligners (CAs) resulted in lingual tipping, extrusion, and distal movement of the central incisor in extraction cases. A labially positioned root amplified lingual tipping and torque loss, whereas a palatally positioned root preserved torque but increased posterior anchorage loss; moreover, shorter roots accentuated tipping and generated peak PDL stresses at the cervical and apical regions, while thinner cortical bone resulted in higher stress.

**Conclusion:**

SRP and RL significantly affect tooth movement and stress distribution during anterior tooth retraction with CAs in extraction cases, while CBT has minimal impact in tooth movement. The optimal pre-retraction state involves a crown-to-root ratio not exceeding 1.1, with the root positioned upright in the cancellous bone, preventing contact with the labial or palatal cortical bone.

## 1 Introduction

Clear aligner therapy (CAT), initially limited to minor crowding or spacing, has expanded to extraction cases through advancements in 3D digital modeling and biomechanical refinement. (Adel et al., 2023). Nonetheless, undesirable tooth movements, such as deepened overbite, lingual inclination of anterior teeth, or mesial tilting of posterior teeth, are unavoidable during extraction space closure. (Dai et al., 2021). Qian et al. observed that, in premolar extraction cases, clear aligners (CAs) demonstrate a more pronounced “roller-coaster effect” compared to fixed orthodontic appliances. (Qian et al., 2024). Control of anterior labiolingual inclination remains challenging, as Dai et al.'s clinical research revealed that central incisors displayed reduced retraction, greater lingual inclination and extrusion than anticipated (Dai et al., 2019). Significant changes in incisor inclination may lead to orthodontic complications, such as dehiscence and fenestration. (Chambrone and Avila-Ortiz, 2021; Bae et al., 2018). Miyama et al. noted that lingual and extrusive incisor movements contribute to a reduction in alveolar bone height, (Miyama et al., 2018), potentially compromising periodontal health and treatment stability. (Yagci et al., 2011).

Finite Element Analysis (FEA) is a noninvasive virtual modeling tool that simulates oral conditions to analyze force dynamics. (Singh et al., 2024). In orthodontics, FEA is widely used to assess tooth movement and stress distribution under various loading conditions. (Knop et al., 2015). Studies using FEA have provided key insights into the biomechanical optimization of extraction cases in CAT. Meng et al. reported that varying power ridge depths had little effect on tooth displacement patterns, but displacement magnitude increased with deeper ridges. (Meng et al., 2023). According to Lyu’s FEA, optimized aligners with differential margin designs and varied thermoplastic thicknesses improved tooth movement efficiency and control during extraction space closure. (Lyu et al., 2023). Mao et al. found that increasing shape designs at extraction space height enhances anterior teeth retraction, while lowering CAES height aids controlled root movement during extraction space closure. (Mao et al., 2024). Semi-ellipsoid attachments proved uperior for labiolingual inclination control, while power ridges enhanced root control, as noted by Hong (2024).

Prior studies have primarily concentrated on enhancing tooth movement efficiency in CAT by refining aligner designs, attachments, and tooth movement strategies. However, patient-specific factors, such as alveolar bone thickness, tooth root morphology, and sagittal skeletal pattern, significantly influence efficiency and constrain movement range. (Coşkun and Kaya, 2019; Rodrigues et al., 2023). For instance, Zhou et al. found that teeth with longer clinical crowns exhibit reduced torque loss during translational movement, whereas shorter crowns are more prone to bodily movement. (Zhou et al., 2025). Similarly, Sun’s FEA of models with varying alveolar bone resorption highlighted the need to minimize stress on periodontal tissues in patients with bone loss to prevent irreversible damage. (Sun et al., 2019). Despite these findings, FEA studies examining how root/cortical bone relation—like root length (RL), cortical bone thickness (CBT), and sagittal root position (SRP) of maxillary anterior teeth—influence tooth movement efficiency and safety are limited. In addition, deformation of the aligner leads to inconsistent contact points with the crown, causing uncertainty in the force’s magnitude, direction, and application point. (Barone et al., 2017; Liu, 2023). The complex biomechanics of clear aligners further complicates predicting anterior tooth retraction in extraction cases across diverse patients.

Therefore, this study aims to evaluate the effects of different root/cortical bone relation (with varying RL, CBT, and SRP) on tooth movement and stress distribution in CAT for extraction cases using finite element analysis.

## 2 Materials and methods

One healthy female volunteer aged 26 years was selected based on the following criteria: (1) Bilateral maxillary first premolars extracted with aligned and leveled maxillary dentition, (2) normal tooth morphology with harmonious crown-root ratio, no root resorption, (3) SRP of the maxillary central incisors in the alveolar bone middle, (3) no significant periodontal soft or hard tissue recession with normal Alveolar bone thickness, (4) no temporomandibular joint disorders. Cone-beam computed tomography (CBCT) data were acquired using a KaVo Dental GmbH scanner (KaVo, United States) under these settings: 120 kVp, 0.4 mm voxel size; scanning time, 8.9 s. Data were saved in “.dicom” format. This study was approved by the Ethics Committee of the Affiliated Stomatological Hospital of Chongqing Medical University [CQHS -REC-2025 (LSNo.083)].

**FIGURE 1:**
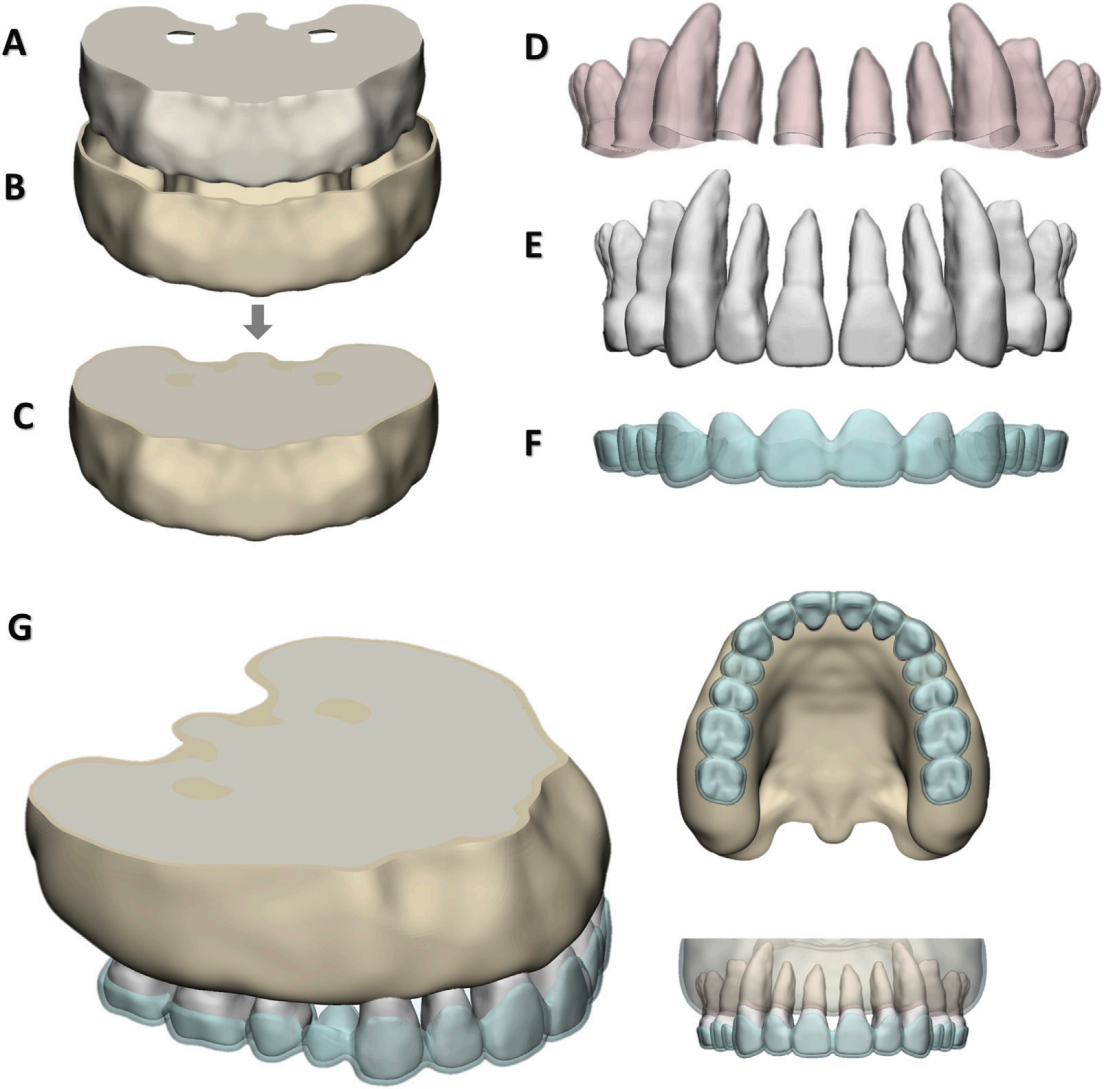
Finite element model: **(A)** cancellous bone; **(B)** Cortical bone; **(C)** Alveolar bone; **(D)** PDL; **(E)** Maxillary dentition; **(F)** CA; **(G)** Final assembly.

A 3-dimensional preliminary model of the maxillary dentition and maxilla was established with CBCT data using Mimics 21.0 (Materialise, Belgium) by adjusting thresholds based on grayscale differences. The model was adjusted and symmetrically aligned along the midsagittal plane to develop a complete maxillary dentition and maxilla model using Geomagic Wrap 2017 (Geomagic, United States) and SolidWorks 2022 (SolidWorks, United States). To create models with varying root lengths for anterior teeth, the root apices of maxillary anterior teeth were reduced by 2 mm and 4 mm, respectively, resulting in three maxillary dentition models. (Figure 1E). After calculation, the crown-to-root ratios, from smallest to largest, were 1.3(long root), 1.1(normal root), and 0.8 (short root). PDL was generated by offsetting each tooth’s surface outward by 0.2 mm using Geomagic Wrap 2017, followed by Boolean operations using SolidWorks 2022 (Figure 1D). The anterior maxillary teeth were retracted sagittally by 0.2 mm, and the updated maxillary dentition was shelled outward to a thickness of 0.75 mm to generate the aligner model using Boolean operations (Figure 1F). The maxilla model was hollowed outward by 1 mm and both outward and inward by 1 mm each (keeping the outer surface dimensions of the cortical bone model consistent, with thicknesses of 1 mm and 2 mm respectively), producing two cortical bone models (Figure 1B). Through Boolean operations, two cancellous bone models were generated from these cortical bone models (Figure 1A). To acquire the alveolar fossa of the maxilla, dentition, and PDL were subtracted from the maxilla by Boolean operation (Figure 1C). Three different SRPs of maxillary anterior teeth were established: (1) created by translating the teeth labially by 2.5 mm from their original position; (2) the original position; and (3) lingually by 2.5 mm. These resulted in three distinct alveolar socket morphologies. All components were assembled to form the final assembly (Figure 1G).

**FIGURE 2:**
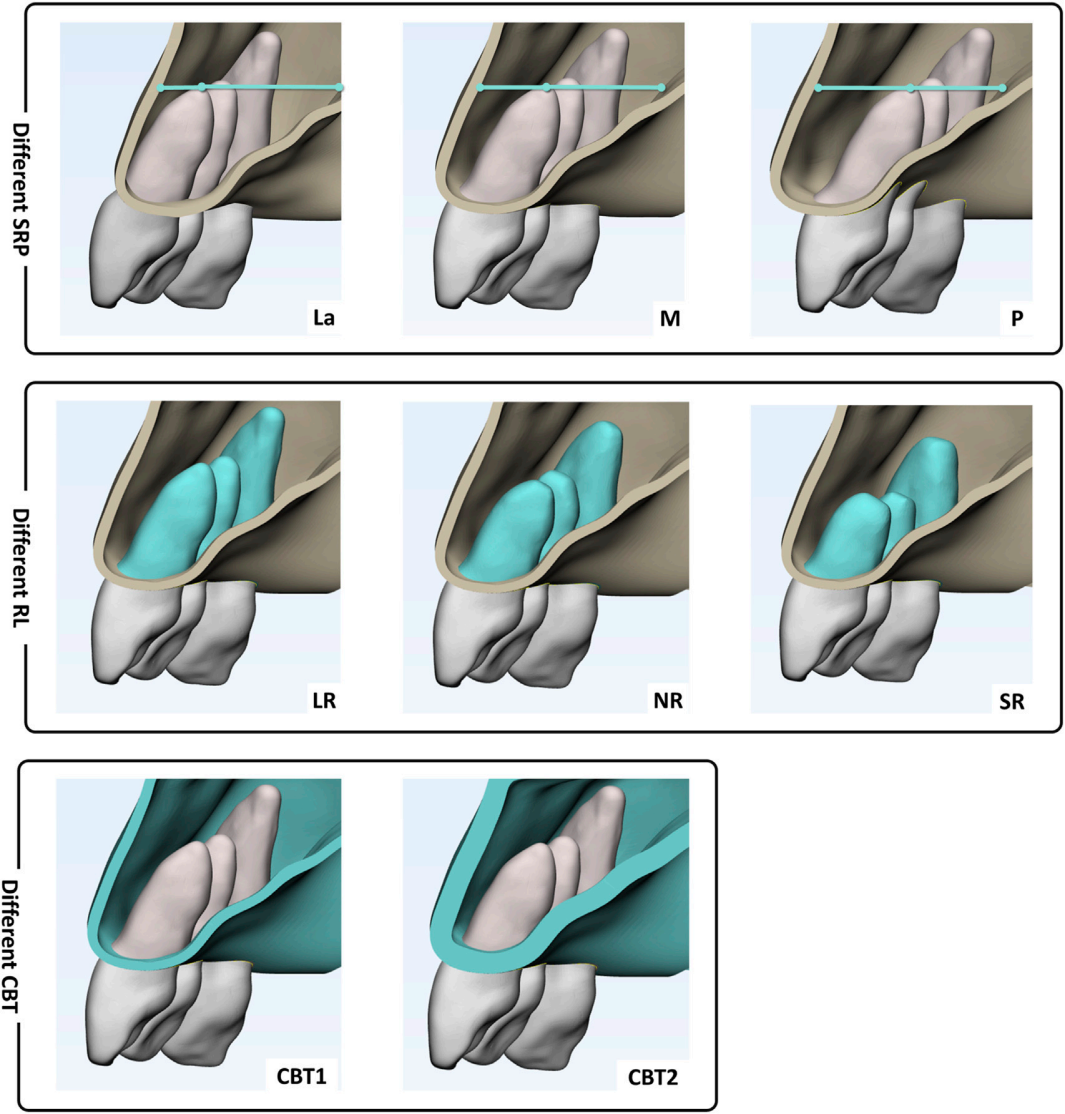
Grouping: Sagittal root position (SRP): on the labial side (La), in the middle (M), on the palatal side (P); Root length (RL): long root (LR), normal root (NR)and short root (SR); Cortical bone thickness of 1 mm (CBT1) and 2 mm (CBT2).

Eighteen model groups were established based on SRP, RL and CBT (Figure 2). The SRP is denoted as La for the labial side, M for the middle, and P for the palatal side. RL is represented as LR for long root, NR for normal root, and SR for short root. CBT is indicated as CBT1 for 1 mm and CBT2 for 2 mm. For example, the group characterized by a sagittal root position on the labial side, long root, and cortical bone thickness of 1 mm is denoted as La-LR-CBT1, and so forth.

The material properties of each component were assumed to be linear elastic, isotropic, and homogeneous. The Young’s moduli and Poisson’s ratios for teeth, alveolar bone (composed of cortical and cancellous bone), the PDL, and the aligner are presented in Table 1. (Davy et al., 1981; Tanne et al., 1987; Liang et al., 2009; Gomez et al., 2015; Wang et al., 2024). Meshing was conducted with mesh sizes of 1.0 mm for teeth, 2.0 mm for alveolar bone (cortical and cancellous), 0.5 mm for the PDL, and 0.6 mm for the aligner (Davy et al., 1981). Node and element counts for the teeth, alveolar bone, PDL, and aligner models in each group are presented in Table 2.

**TABLE 1:** Material properties.

Component	Young’s Modulus	Poisson’s ratios
Teeth	19,600 MPa	0.30
Cortical bone	13,700 MPa	0.30
Cancellous bone	1,370 MPa	0.30
PDL	0.69 MPa	0.45
Aligner	528 MPa	0.36

**TABLE 2:** Number of nodes and elements of the groups of the finite element model.

Group	Nodes	Elements
LA-LR-CBT1	477254	270148
LA-LR-CBT2	474938	269019
M-LR-CBT1	481469	272339
M-LR-CBT2	477905	270508
P-LR-CBT1	480440	271629
P-LR-CBT2	477533	270200
LA-NR-CBT1	511113	288004
LA-NR-CBT2	509763	287453
M-NR-CBT1	517910	291586
M-NR-CBT2	514469	289779
P-NR-CBT1	518352	291622
P-NR-CBT2	515488	290261
LA-SR-CBT1	496732	280326
LA-SR-CBT2	493832	279035
M-SR-CBT1	502919	283552
M-SR-CBT2	499570	281827
P-SR-CBT1	502557	283164
P-SR-CBT2	499438	281515

Contact settings and boundary conditions were implemented. The maxillary alveolar bone base was fixed, with bonded contacts set between teeth and PDL, PDL and alveolar bone, and cortical and cancellous bone. Frictional contact, with a 0.2 friction coefficient, was defined between the outer crown surface and the inner aligner surface. No further constraints or loads were applied to the teeth or aligner.

A global coordinate system was established: the X-axis (coronal direction) aligned parallel to the plane, directed toward the patient’s right (positive) or left (negative); the Y-axis (sagittal direction) set perpendicular to the X-axis, oriented toward the central incisor (positive) or opposite (negative); and the Z-axis (vertical direction), perpendicular to both X and Y-axes, directed apically (positive) or coronally (negative).

Finite element analysis (FEA) was conducted using Ansys 2024 (Ansys, United States) for calculations. The analysis included: (1) initial tooth movement tendencies and displacement in the X-, Y-, and Z-axes.; (2) maximum von Mises stresses in the PDL and alveolar bone.

## 3 Result

**FIGURE 3:**
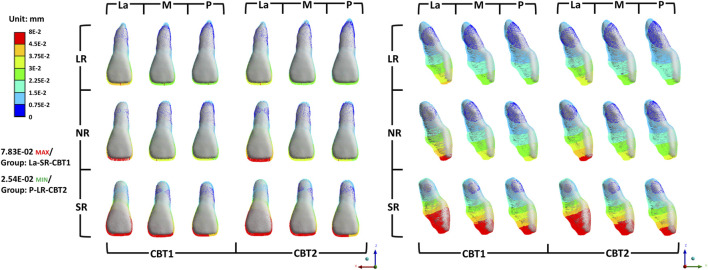
The initial tooth movement tendencies of maxillary central incisor with varying SRP/RL/CBT in coronal and sagittal directions.

In all groups, the maxillary central incisors exhibited opposing crown and root movements. The crowns moved distally and lingually with extrusion and slight distal-lingual torsion, while the roots moved mesially and labially with intrusion (Figure 3). SRP and LR significantly influenced tooth movement, whereas CBT had no notable effect (P > 0.05).

**FIGURE 4:**
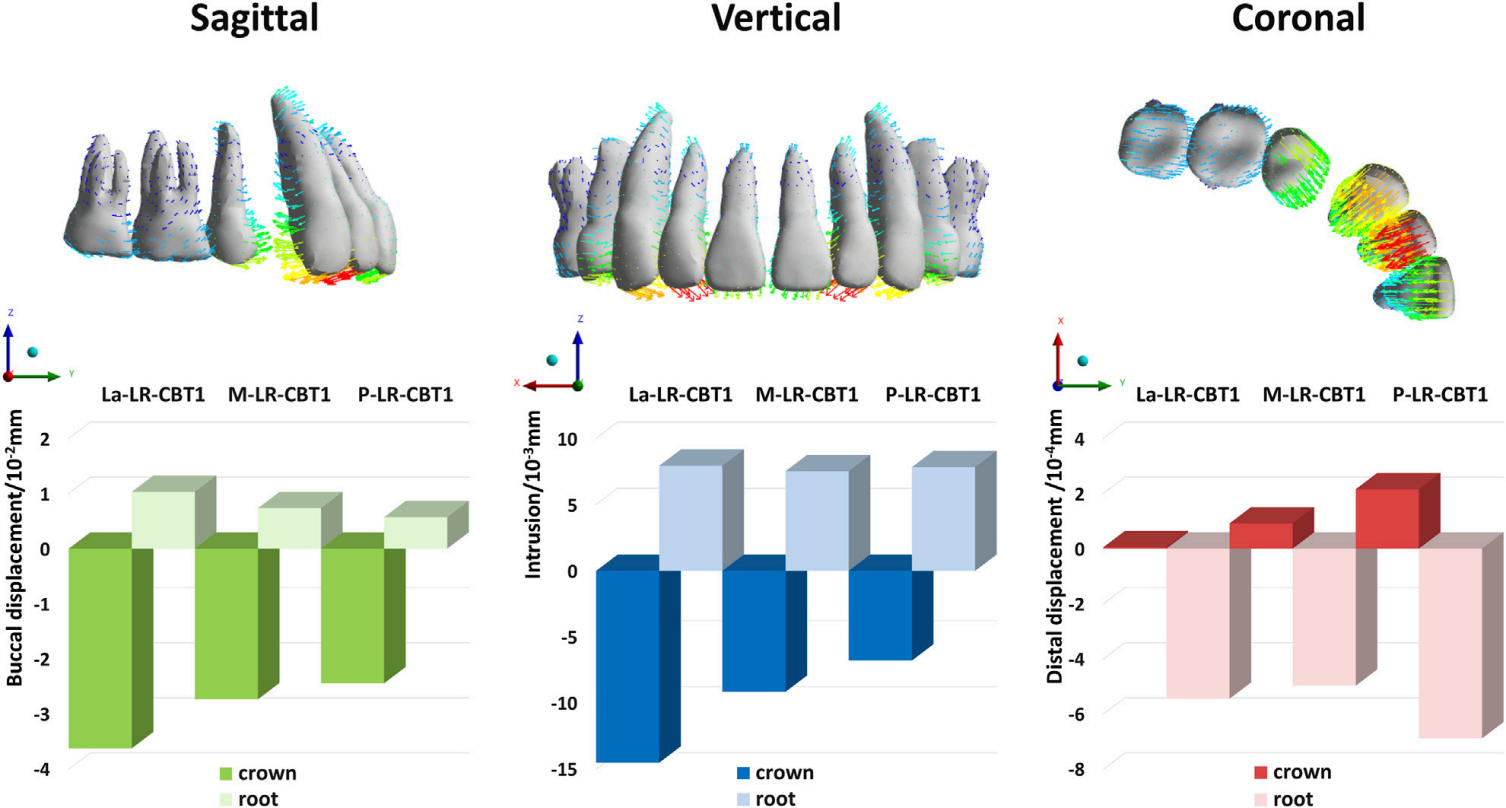
Bar charts: Three-dimensional displacement of the crown and root of maxillary central incisors under different SRP (with LR, CBT1).

**FIGURE 5:**
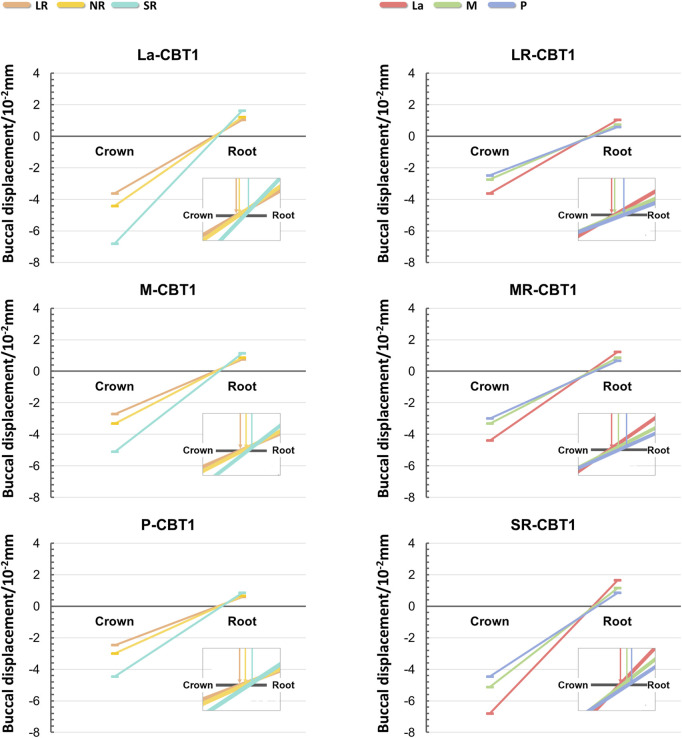
Line charts: Displacement of the crown and root of the maxillary central incisor under different SRP and RL (with CBT1). The point where the line intersected the x-axis marked the center of rotation, and the slope reflected the shift in labiolingual tilt.

Changes in the three-dimensional direction were predominantly sagittal (Figure 4). When SRP varied, the La groups demonstrated the poorest torque control, with greater sagittal and vertical displacement of the root and apex in the opposite direction (Figures 4, 5). In the coronal direction, the P groups showed the largest axial inclination change. As RL decreased, torque control deteriorated, leading to a more pronounced lingual tipping of the maxillary central incisors (Figure 5). For groups with different CBTs, differences between 1 mm and 2 mm were minimal (Figure 3).

**FIGURE 6:**
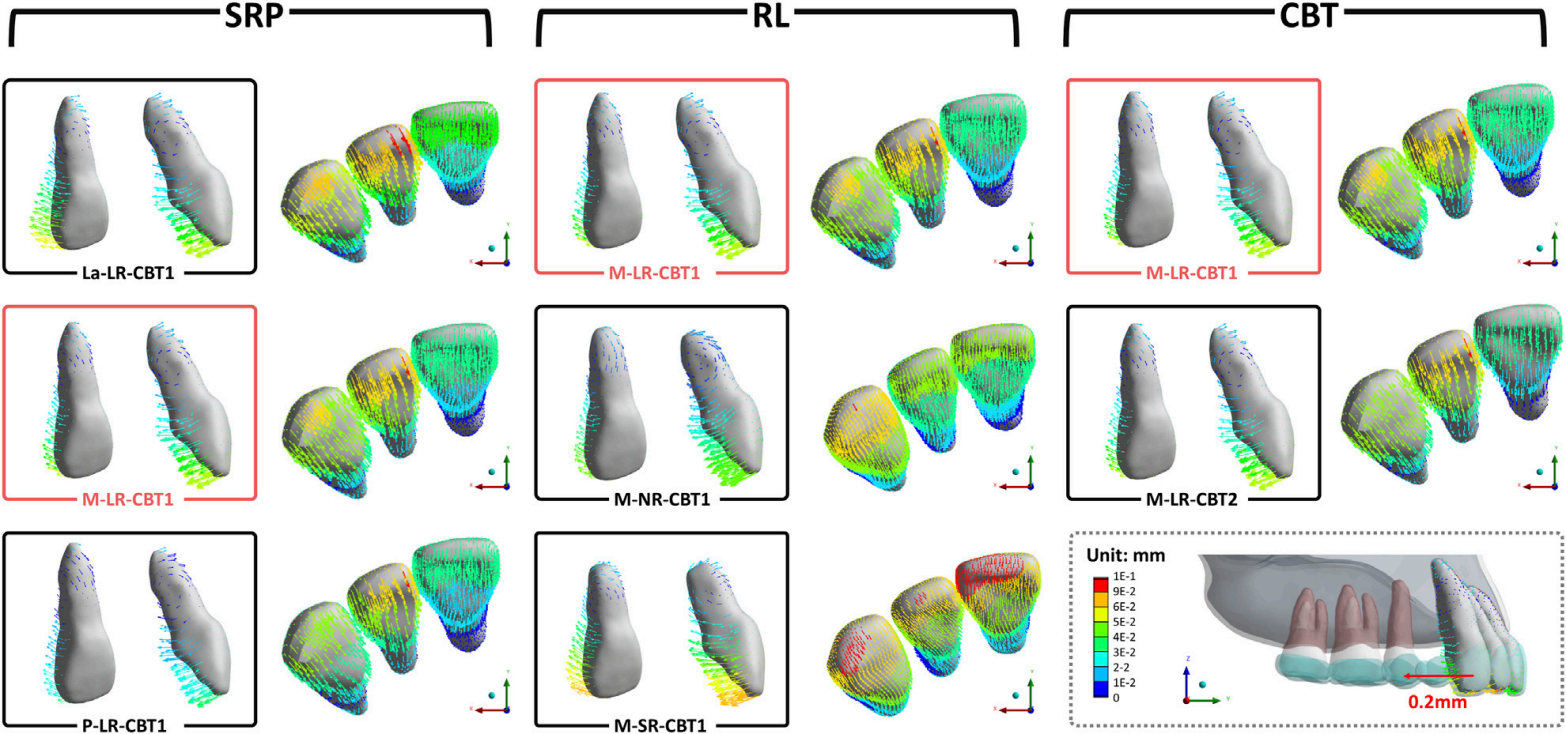
The initial tooth movement tendencies of anterior teeth with varying SRP/RL/CBT in occlusal directions. (M-LR-CBT1 as the control group).

Lateral incisors and canines exhibited similar movement tendencies to central incisors (Figure 6). Using M-LR-CBT1 as the control group, the effects of varying SRP, RL, and CBT on anterior teeth movement are illustrated in Figure 6.

**FIGURE 7:**
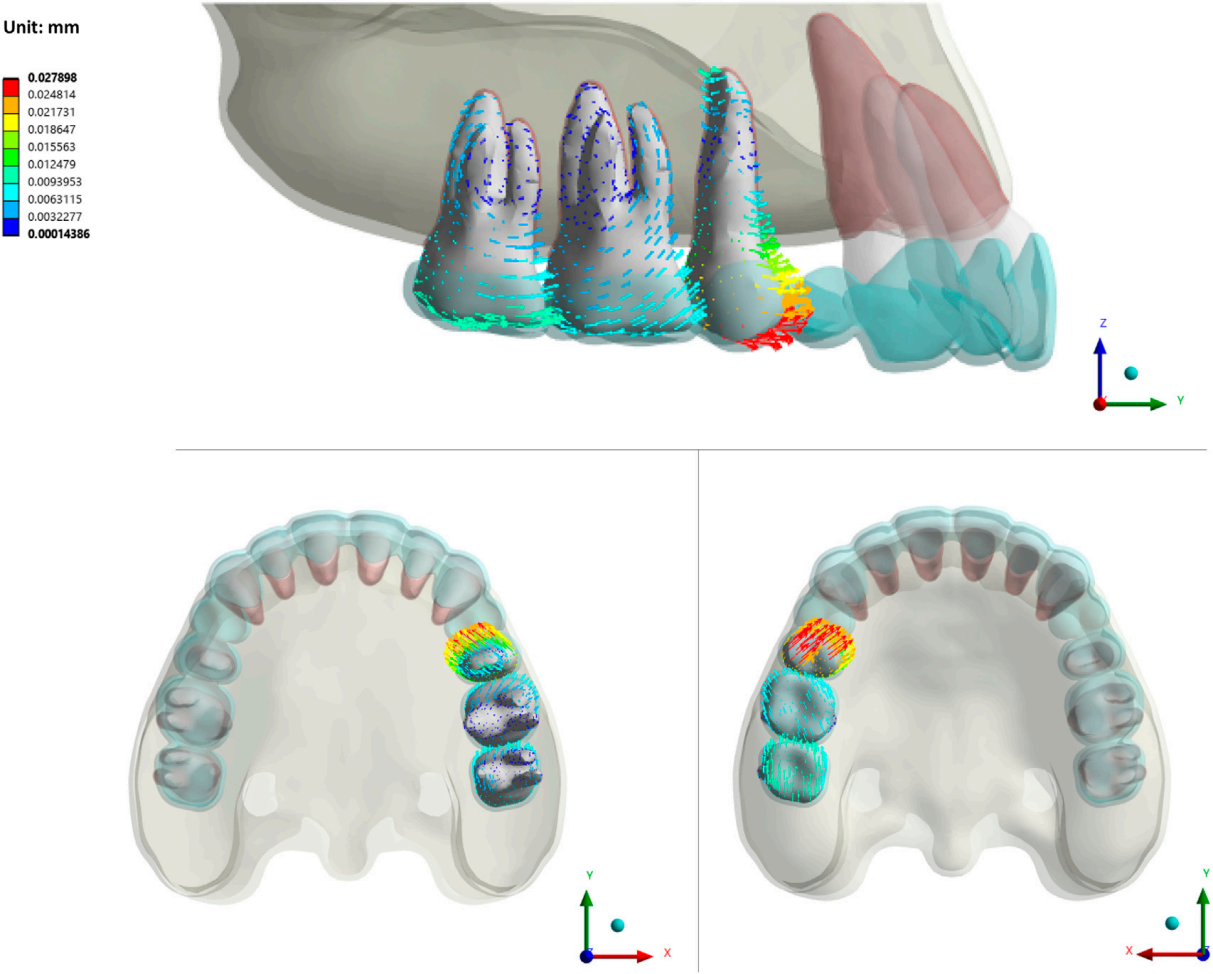
The initial tooth movement tendencies of posterior teeth with varying SRP/RL/CBT in sagittal/apex/occlusal directions (La-LR-CBT1).

Although the posterior teeth were not designed to move, they all exhibited a tendency for the crowns to move mesially and the roots to move distally in all groups (Figure 7)

**FIGURE 8:**
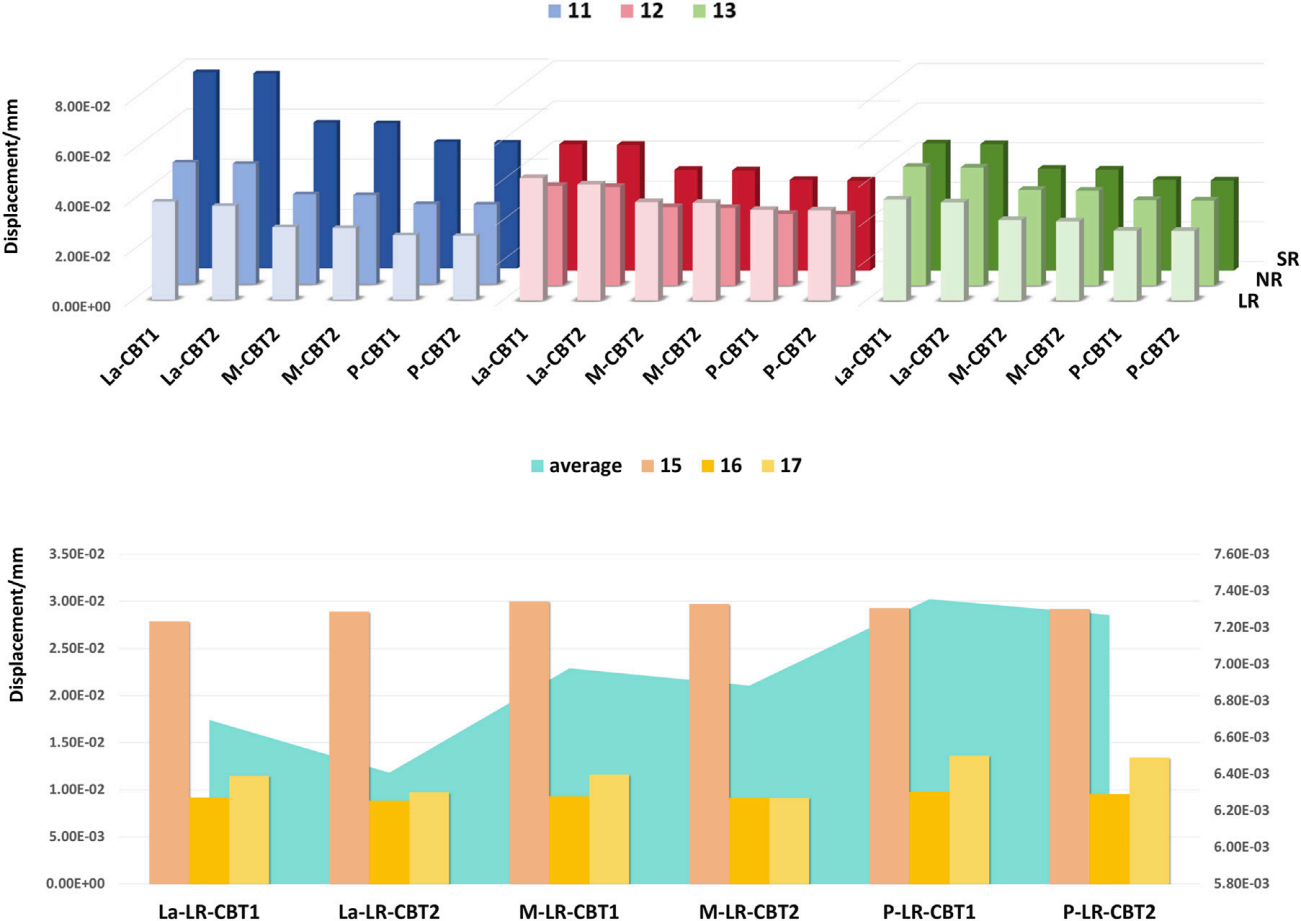
Bar charts: displacement of each tooth with varying SRP/RL/CBT.

The maximum displacement of each tooth is presented in Figure 8. Among anterior teeth, lateral incisors exhibited the greatest displacement, while central incisors showed the least. Among posterior teeth, second premolars showed the greatest mesial crown inclination, while first molars showed the least. Using average posterior tooth displacement to represent posterior anchorage loss, the P groups exhibited the greatest anchorage loss. Overall, anterior teeth displayed greater displacement than posterior teeth.

**FIGURE 9:**
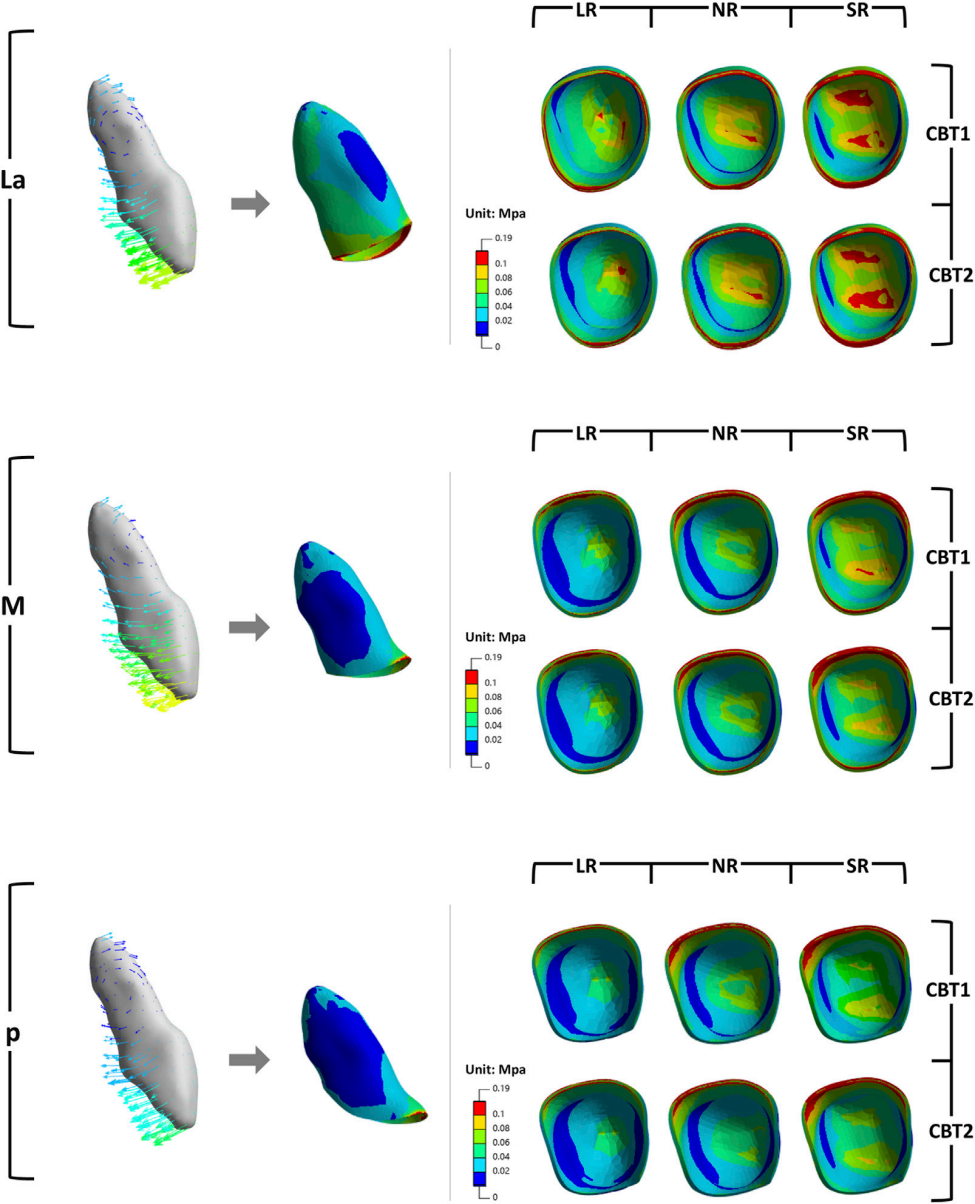
Von Mises stress values of PDL of the maxillary central incisor.

Von Mises stress concentrations were observed in the cervical areas and root apices of the periodontal ligament (PDL) of all central incisors, aligning with tooth movement patterns (Figure 9). The M groups exhibited the lowest stress, followed by the P groups, with the La groups showing the highest. Within each group, shorter roots were associated with progressively higher Von Mises stress, while increased cortical bone thickness significantly reduced stress (P < 0.05).

**FIGURE 10:**
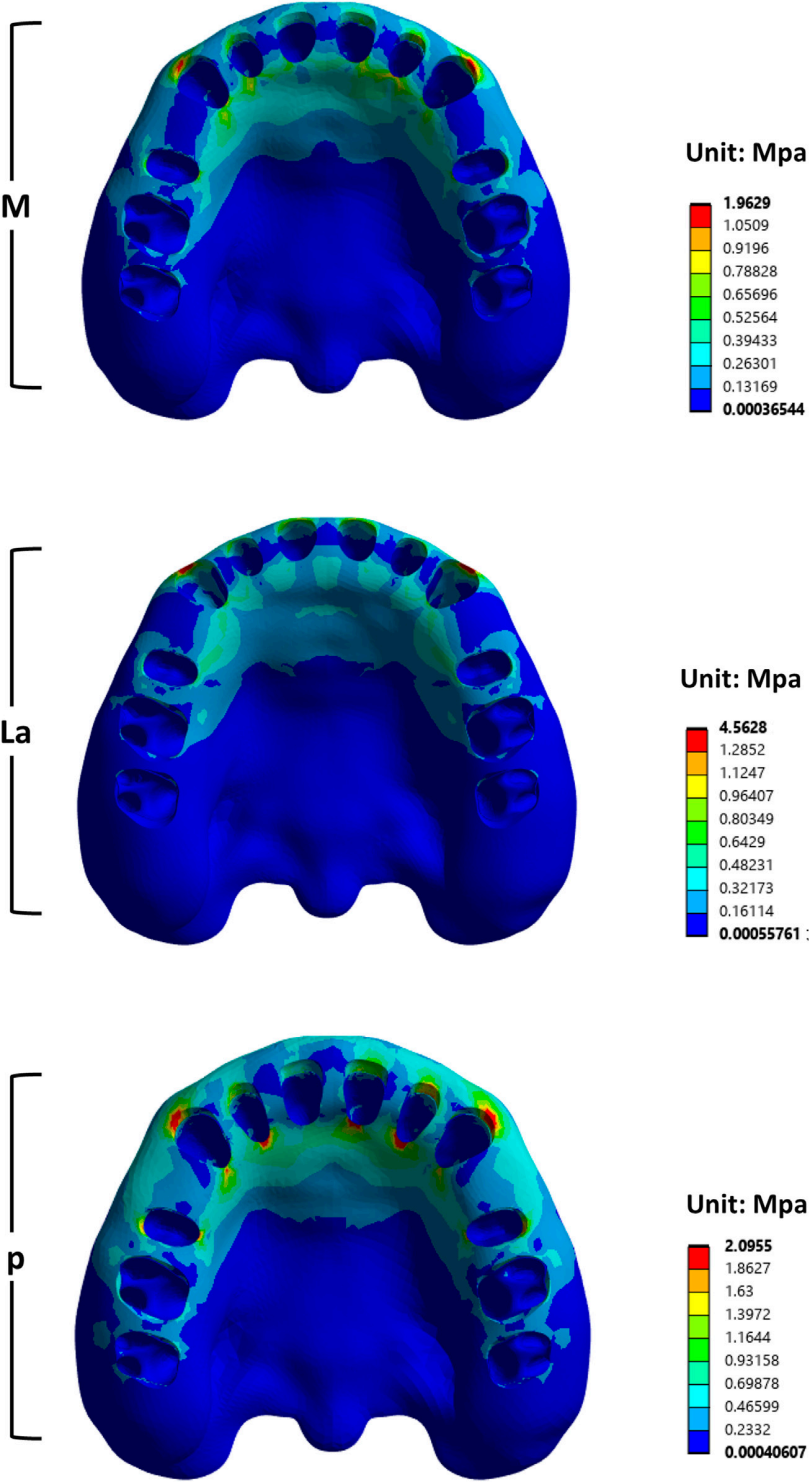
Von Mises stress values of maxillary alveolar bone under different SRP.

For the alveolar bone, stress was significantly concentrated on the labial alveolar wall of the canine (Figure 10). The La groups showed the highest stress among the three.

## 4 Discussion

This FEA found that during anterior tooth retraction using CAs without attachments or additional forces, teeth exhibited tilting with opposing crown and root movements. Maxillary central incisors demonstrated lingual tipping, extrusion, and slight distal movement, with lateral incisors and canines showing similar patterns. The inclination of anterior teeth results from the resultant force of CAs missing the center of resistance (CR), generating rotational moments. (Theodorou et al., 2019; Geramy et al., 2022; Jacob et al., 2024). The CR of maxillary central incisors is located approximately 6–8 mm apical to the crest along the tooth’s long axis, as confirmed by Viecilli et al. through 3D analysis. (Viecilli et al., 2013). However, CAs envelop the crown, applying forces far from the CR, generating moments. (Elshazly et al., 2022; Li et al., 2024; Aminian et al., 2024). Consequently, bodily retraction of anterior teeth in extraction cases is nearly unachievable using CAs. (Kang et al., 2023). Previous FEA studies identified a roller-coaster effect in the maxillary and mandibular arches during extraction space closure and reported significant lingual inclination and extrusion of anterior teeth without power ridge designs. (Liu, 2023; Meng et al., 2023).

**FIGURE 11:**
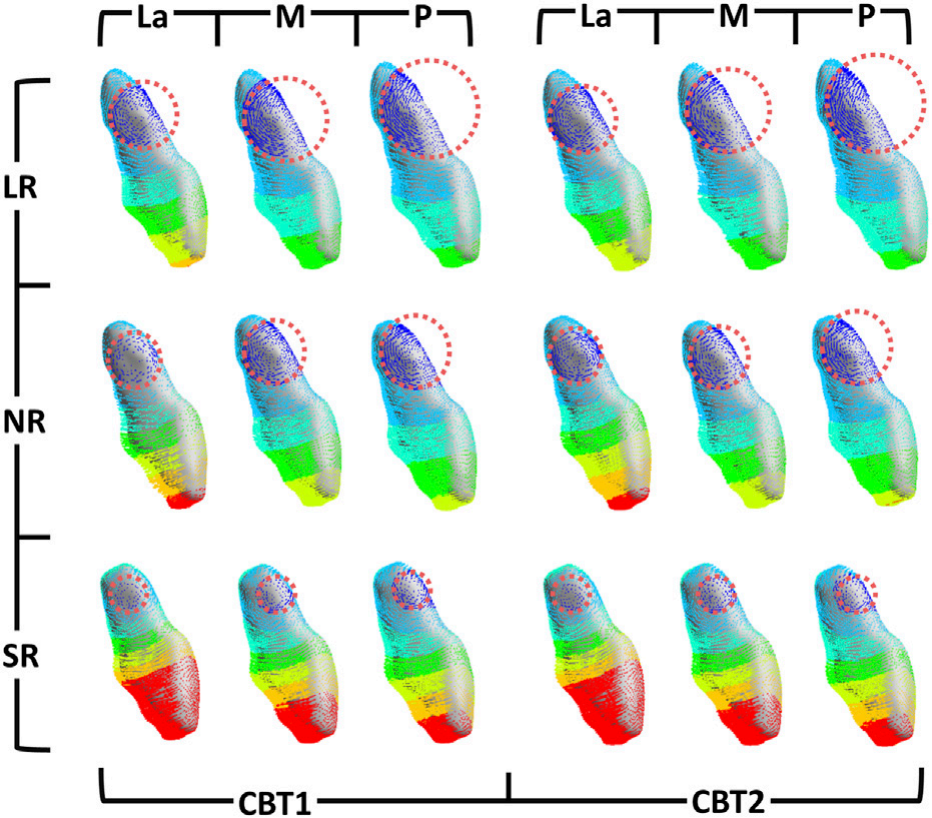
Mesial view of the initial tooth movement tendencies of the maxillary central incisor with varying SRP/RL/CBT. Rotation centers were approximated by visual best-fit.

Furthermore, among groups with different sagittal root positions (SRPs), the La groups exhibited the most pronounced anterior tooth tilting. Due to minimal labial bone resistance, labial crown movement exceeded root apex movement, resulting in greater retraction (Figure 11). The P groups showed minimal anterior tooth movement and optimal torque control, due to palatal cortical bone anchorage. Ricketts notes that dense cortical bone provides robust anchorage, with root contact against cortical bone enhancing stability. (Ricketts, 1976). The M groups displayed tilting similar to the P groups but with slightly inferior anterior anchorage control, both outperforming the La groups. Variations in SRP ultimately altered the rotation axis, causing variable displacement. A more labial SRP shifts the sagittal rotation center closer to the palatal side, as shown in Figure 11.

Posterior teeth, although not intended to move, displayed mesial crown tipping and distal root tipping. Mesial movement in posterior teeth was less than distal movement in anterior teeth due to larger root surface area, indicating moderate molar anchorage in extraction cases. Among posterior teeth, the second premolar exhibited the most pronounced mesial crown tipping, while the first molar showed the least. This was due to inadequate aligner rigidity at the extraction site, which compromised force transmission and shape retention during retraction. (Dai et al., 2021; Upadhyay and Arqub, 2022). Among groups with different SRPs, the P groups experienced the greatest posterior anchorage loss, characterized by mesial movement and crown tipping. This was consistent with the finding that the P groups showed minimal anterior tooth displacement.

Shorter root led to greater tilting. Figure 11 illustrates vertical changes in the rotation center of maxillary central incisors with varying RLs across groups. Similarly, Figure 5 shows the SR group’s rotation axis line intersecting the horizontal axis closest to the root, indicating a rotation axis nearest the apex. Prior research has shown that in short-rooted teeth, the center of resistance is nearer to the crown, increasing the likelihood of uncontrolled moments from applied forces (Jinrui et al., 2023). With the center of resistance closer to the crown in short-rooted teeth, the rotation axis shifts toward the apex, heightening the risk of lingual tipping and crown extrusion during retraction.

This study modeled CBTs of 1 mm and 2 mm, controlling for consistent alveolar bone thickness. Although CBT did not significantly affect the magnitude of initial tooth displacement in this model, it functioned as a critical mechanical boundary condition. A trend was observed whereby thinner cortical bone (1 mm) increased periodontal ligament stress. However, our positive findings were derived from anterior retraction under two CBT conditions. Consequently, these results should not be extrapolated to other tooth movements—such as extrusion, intrusion, or torque correction—where root-to-cortex proximity may exert a far greater influence. At present, the effect of CBT on these unexplored types of tooth movement remains essentially unknown and must be investigated under dynamic or prolonged loading.

Von Mises stress quantifies PDL stress distribution, revealing stress concentrations. Excessive stress may cause PDL ischemia and root resorption, while insufficient stress reduces movement efficiency. (Li et al., 2021). This study found stress concentrations at the root apices and cervical regions of anterior teeth in all groups, most notably in the La groups. The La groups exhibited the highest von Mises stress due to greater root displacement compared to other groups. The P groups showed high PDL stress due to resistance from palatal cortical bone, restricting movement. Movement within cancellous bone, the M groups’ pattern, reduced root resorption risk. Shorter roots increased von Mises stress, as shown in Figure 8. In short-rooted teeth, a 1:0.8 crown-root ratio increases tipping and root resorption risk compared to 1:1.1 and 1:1.3 ratios, requiring slower retraction and intrusion to prevent bone fenestration or dehiscence.

In clinical practice, to effectively close extraction spaces, orthodontists must maintain proper incisor torque and molar anchorage control to achieve an optimal cusp-fossa relationship. (Zhu et al., 2023). Molar anchorage requirements vary among orthodontic patients, based on both the ideal sagittal positions of the incisors and the patient’s dental and periodontal conditions.

Cases requiring substantial retraction typically necessitate strong molar anchorage designs to prevent anterior torque loss and maintain the sagittal position of posterior teeth. This study found that the optimal pre-retraction state is a crown-root ratio not exceeding 1.1 (the NR group), with roots upright in cancellous bone and not contacting labial or palatal cortical bone. While this ideal condition may not be achievable in all patients, orthodontists can optimize outcomes through personalized designs. For patients with a labial SRP, maintaining anterior torque is the primary challenge during retraction. These cases often show poor predictability, particularly with pre-existing lingual tipping or deep overbite. Clinical strategies include applying additional torque, stepwise and overcorrected intrusion designs, or using retention attachments may prevent lingual tipping and deep overbite during extraction space closure. (Zhang et al., 2024; Kang et al., 2023; Liu, 2023). However, reduced labial alveolar bone resistance may increase the risk of bone fenestration or dehiscence, necessitating precise force control. (Li et al., 2025; Sun et al., 2021). Excessive initial stress in aligners should be managed cautiously to avoid exceeding cortical bone limits. (Jacob et al., 2024; Upadhyay and Arqub, 2022). For patients with a palatal SRP, strong posterior anchorage designs are unsuitable, as maintaining the sagittal position of posterior teeth is challenging. Anterior cortical bone anchorage increases periodontal ligament stress on anterior teeth when posterior anchorage is excessively enhanced, elevating root resorption risk.

In cases with minimal or moderate anchorage, posterior teeth may move mesially, but mesial tipping should be prevented. Strategies to prevent posterior mesial tipping include Class II traction, power arms, or proactive anchorage preparation. (Qiang et al., 2025; Ahmed et al., 2019; Wang et al., 2022). If significant posterior tipping occurs, auxiliary techniques such as sectional arches may be necessary. (Caldas et al., 2014). The primary focus in these cases is to prevent anterior tooth fenestration, dehiscence, and root resorption. Contact between anterior roots and palatal cortical bone should be avoided, especially when the SRP is palatally located. Using palatal cortical bone as anchorage is not recommended, even in cases with minimal or moderate anchorage.

For patients with shorter roots, orthodontists should evaluate baseline conditions such as SRP. This helps determine whether extraction treatment is suitable, minimizing risks of torque loss, anchorage failure, and further root resorption. Although PDL stress in short-rooted teeth remains below clinical limits in this study, orthodontists should reduce step distances, or adjust power ridge depth to minimize resorption.

Moreover, Patients with thinner cortical bone may experience more pronounced undesired tooth movement and higher periodontal-ligament stresses; consequently, smaller step distances are required to prevent torque loss, bone fenestration, or dehiscence.

It should be noted that in clinical practice, the condition of patients’ tooth roots and alveolar bone may change between initial diagnosis and after the stage of alignment and leveling. To ensure safe tooth movement within the alveolar bone, the position of tooth roots within the alveolar bone must be evaluated before anterior retraction. (Barootchi et al., 2020; Rekhi et al., 2020). Clinical examination can confirm the sagittal root position, alveolar bone thickness, and presence of root resorption, with cone-beam computed tomography (CBCT) imaging employed when necessary.

Limitations: This FEA study has limitations. The complexity of tooth geometry and periodontal tissues makes simulating aligner-tooth interactions challenging, with variations across individuals and teeth. The model does not address biological processes like PDL changes or bone and gingival remodeling This static FEA captures only immediate stress, not the cumulative, time-dependent root-resorption risk that arises from prolonged loading and individual biology. Validation through animal or clinical studies is necessary, as FEA alone is insufficient. Future research should develop optimized FEA models and conduct clinical validations.

## 5 Conclusion

In this finite element analysis study, the effects of different root/cortical bone relation (with varying RL, CBT, and SRP) on anterior tooth retraction in clear aligner therapy (CAT) with premolar extraction were evaluated. The following conclusions were drawn:

1. Premolar extraction space closure via CAT caused distal and lingual movement with extrusion of maxillary central incisors.
2. SRP and RL significantly affect tooth movement and stress distribution during anterior tooth retraction with CAs in extraction cases, while CBT has minimal impact in tooth movement.
3. The ideal pre-retraction condition is a crown-to-root ratio no more than 1.1, with the root upright in the cancellous bone, avoiding contact with the labial or palatal cortical bone.
4. For patients with SRP on the labial side, additional labial torque on anterior teeth is required to prevent bone fenestration or dehiscence.
5. For patients with SRP on the palatal side, care should be taken to prevent root resorption due to excessive stress on PDL of anterior teeth.

## Data Availability

The original contributions presented in the study are included in the article/Supplementary Material, further inquiries can be directed to the corresponding authors.

## Abbreviations

CBCT, Cone-beam computed tomography; CAT, clear aligner treatment; CA, clear aligner; PDL, periodontal ligament; FEA, Finite Element Analysis; SRP, sagittal root position, RL, root length, CBT, cortical bone thickness.
