# Supplementary material for: Biomechanical effects of root/cortical bone relation on tooth movement during premolar-extraction space closure with clear aligners: a finite element study
Source: Front Bioeng Biotechnol. 2025 Nov 19;13:1717813. doi: 10.3389/fbioe.2025.1717813 (PMC12672540; doi:10.3389/fbioe.2025.1717813)
Supplement: Supplementary file 1 [file Table1.docx]

Table III. Maximum displacement (mm) of the anterior teeth and posterior teeth

| Group | 11 | 12 | 13 | 15 | 16 | 17 |
| --- | --- | --- | --- | --- | --- | --- |
| LA-LR-1 | 3.91E-02 | 6.11E-02 | 5.10E-02 | 2.79E-02 | 9.19E-03 | 1.15E-02 |
| LA-LR-2 | 3.74E-02 | 5.79E-02 | 4.96E-02 | 2.89E-02 | 8.82E-03 | 9.75E-03 |
| M-LR-1 | 2.89E-02 | 4.91E-02 | 4.06E-02 | 3.00E-02 | 9.30E-03 | 1.16E-02 |
| M-LR-2 | 2.86E-02 | 4.86E-02 | 4.00E-02 | 2.97E-02 | 9.17E-03 | 9.17E-03 |
| P-LR-1 | 2.57E-02 | 4.52E-02 | 3.52E-02 | 2.93E-02 | 9.78E-03 | 1.36E-02 |
| P-LR-2 | 2.54E-02 | 4.50E-02 | 3.51E-02 | 2.92E-02 | 9.55E-03 | 1.34E-02 |
| LA-NR-1 | 4.85E-02 | 4.97E-02 | 6.00E-02 | 2.94E-02 | 8.16E-03 | 9.42E-03 |
| LA-NR-2 | 4.80E-02 | 4.92E-02 | 5.96E-02 | 2.93E-02 | 8.06E-03 | 9.25E-03 |
| M-NR-1 | 3.58E-02 | 3.91E-02 | 4.83E-02 | 2.98E-02 | 8.82E-03 | 1.12E-02 |
| M-NR-2 | 3.55E-02 | 3.87E-02 | 4.79E-02 | 2.98E-02 | 8.66E-03 | 1.10E-02 |
| P-NR-1 | 3.20E-02 | 3.57E-02 | 4.32E-02 | 3.03E-02 | 9.21E-03 | 1.33E-02 |
| P-NR-2 | 3.18E-02 | 3.55E-02 | 4.29E-02 | 3.01E-02 | 8.99E-03 | 1.30E-02 |
| LA-SR-1 | 7.83E-02 | 6.30E-02 | 6.43E-02 | 2.51E-02 | 6.60E-03 | 7.86E-03 |
| LA-SR-2 | 7.79E-02 | 6.27E-02 | 6.39E-02 | 2.50E-02 | 6.50E-03 | 7.72E-03 |
| M-SR-1 | 5.83E-02 | 5.03E-02 | 5.15E-02 | 2.58E-02 | 7.31E-03 | 9.59E-03 |
| M-SR-2 | 5.80E-02 | 5.00E-02 | 5.11E-02 | 2.56E-02 | 7.22E-03 | 9.56E-03 |
| P-SR-1 | 5.05E-02 | 4.53E-02 | 4.59E-02 | 2.66E-02 | 7.79E-03 | 1.17E-02 |
| P-SR-2 | 5.03E-02 | 4.49E-02 | 4.55E-02 | 2.66E-02 | 7.64E-03 | 1.15E-02 |
